# Supplementary material for: Metabolism and Health Effects of Rare Sugars in a CACO-2/HepG2 Coculture Model
Source: Nutrients. 2022 Jan 30;14(3):611. doi: 10.3390/nu14030611 (PMC8839664; doi:10.3390/nu14030611)
Supplement: Supplementary file 1 [file nutrients-14-00611-s001.zip › nutrients-1563314-supplementary.pdf]

Mitochondrial & DNA damage

Inflammation

Diabetes, glucose homeostasis and insulin resistance

Diabetes pathology

Lipid metabolism

Table S1a

Genes	Glucose	Fructose	L-arabinose
TXNIP	16.8	2.71	
WFDC2	7.06		
ARRDC4	5.50		
AADACP1	3.86		
TNNI3	3.43		
SLC28A1	3.43		
ZNF441	3.41		
DOK1	2.87		
UGT2B10	2.83		
MAGEH1	2.83		
TMEM229B	2.83		
EXOC3L4	2.77		
CRYM	2.75		
AP001059.1	2.69		
OSBP2	2.62		
ZNF100	2.51		
FZD4	2.41		
TNNT1	2.38		
TFF3	2.36		
COL2A1	2.22		
GPCPD1	2.17		
TRIM59	2.17		
KBTBD7	2.16		
SLC25A47	2.16		
ENSG00000273361	2.14		
AGPAT2	2.14		
MEGF8	2.04		
BRI3BP	2.04		
FBXL16	2.03		
AC132872.1	2.01		
CDKN2C	2		
RPS10-NUDT3	18.5		
FOS	9.32		
WNT7B	6.63		
SOX7	5.31		

BHLHE40	4.89		2.33
ACTA1	4.82		
PLCXD3	4.69		
SCHIP1	4.66		
NGFR	4.56		
DUSP5	4.50		2.14
GNAI1	4.29		
EGR1	3.73		
JDP2	3.66		
NUAK1	3.51		
AC026202.2	3.51		
IL11	3.39		
GPRC5A	3.34		
HSPA1B	3.10		
AKR1B10	2.97		
CCN1	2.91		
FAM110C	2.91		2.57
MISP	2.69		
ANXA3	2.66		
ITPRIP	2.62		
DNAJB4	2.62		
LYVE1	2.60		
METRNL	2.55		
NUPR1	2.55		
PALLD	2.53		
KLF5	2.50		
UBASH3B	2.46		
HERPUD1	2.41		
DUSP1	2.39		
FGD6	2.39		
DDIT4	2.38		2.31
MAFF	2.38		
EPHA2	2.36		
ADM	2.35		2.66
CITED2	2.35		2.04
PPP1R15A	2.28		
GRB10	2.25		
SPRY4	2.25		
DNAJB1	2.25		
ATG14	2.23		
DNAJB9	2.19		
IER3	2.17		
MXD1	2.17		
FBLIM1	2.14		
TANC1	2.13		

STK26	2.08		
CDR2L	2.08		
C9orf152	2.08		
ARHGEF2	2.07		
SOCS5	2.07		
GDF15	2.04		
SLC4A11	2.03		
KDM7A	2.03		
RASD1	2		
ANGPTL4		2.77	3.41
TCIRG1		2.62	
RTN4R		2.30	
AL391416.1		4.63	
GAPDHP35		4.06	3.41
ASNS		2.85	
SLC7A11		2.48	
ADM2		2.35	
SLC6A9		2.14	
SIK1B			2.93
ERRFI1			2.62
ZNF628			2.60
HAMP			2.57
HILPDA			2.36
ENSG00000125968			2.28
TNS4			2.25
INHBB			2.22
PFKFB3			2.17
RN7SL1			2.16
KCNG3			2.14
RPS15P4			2.08
A4GALT			2.08
KLF10			2.08
ARRDC3			2.08
ANKRD37			2.08
ELF3			2.06
LIN54			**2.01**
RIT1			2.01
FAM86EP			3.34
SRGAP3			3.07
NEXN			2.69
BISPR			2.43
AL035461.2			2.31
AC022075.1			2.28

S1b

Genes	Glucose vs Fructose	Glucose vs Mannitol	Fructose vs Mannitol
TXNIP	6.19	16.8	2.71
WNT7B	5.31	6.63	
EGR1	2.97	3.73	
DDIT4	2.69	2.38	
FOSL2	2.32		
FBLIM1	2.11	2.14	

S1c

Genes	Fructose vs L-Arabinose	Fructose vs Mannitol	L-arabinose vs Mannitol
NBPF8	4.66		
FOS	3.01		
BHLHE40	2.77		2.33
DUSP1	2.48		
ERRFI1	2.23		2.62
PPP1R15A	2.22		
RASD1	2.10		
DDIT4	2.03		2.31
RSRC2	2.01		
TIPARP	2		

S1d

Genes	Glucose vs L-arabinose	Glucose vs Mannitol	L-arabinose vs Mannitol
RPS10-NUDT3	25.8	18.5	
FOS	16.7	9.32	
TXNIP	14.7	16.8	
EGR4	11.8		
BHLHE40	11.4	4.89	2.33
DUSP5	9.71	4.50	2.14
RN7SK	9.71		
ACTA1	8.75	4.82	
RPL17-C18orf32	8.28		
FAM110C	7.36	2.91	2.57
DLX3	7.36		
AHHR	7.06		
PFKFB3	7.01		2.17
SIK1B	6.96		2.93

AC013394.1	6.45		
SOX7	6.32	5.31	
ADM	6.28	2.35	2.66
SLC01B3	5.94		
UGT2B11	5.74		
DDIT4	5.58	2.38	2.31
RPS26P19	5.58		
EGR1	5.39	3.73	
GNAI1	5.28	4.29	
ENSG00000260788	5.21		
ENSG00000224945	5.17		
BANCR	5.10		
SLC28A1	5.03	3.43	
ENSG00000263244	4.96		
CITED2	4.82	2.35	2.04
AL360012.1	4.79		
SCHIP1	4.66	4.66	
CCN1	4.59	2.91	
PPP1R15A	4.50	2.28	
DUSP1	4.50	2.39	
CYP1A1	4.47		
NGFR	4.35	4.56	
KLF5	4.32	2.50	
SOCS3	4.32		
GPRC5A	4.29	3.34	
ACSBG1	4.23		
ENSG00000251867	4.14		
DUSP4	4.06		
LRRC25	4		
DUXAP9	3.94		
PSME2P2	3.92		
ZFP36	3.89		
FRY-AS1	3.86		
TNS4	3.86		2.25
NOCT	3.86		
ATOH8	3.84		
FOSL2	3.76		
ANXA3	3.76	2.66	
ANKRD37	3.76		2.08
F2RL1	3.73		
ACSM2A	3.73		
TPM3P9	3.71		
ELF3	3.71		2.06
MAFF	3.71	2.38	
ALDH3A1	3.63		

CXCR4	3.61		
SLC16A6	3.58		
RNU1-28P	3.58		
RNU1-27P	3.58		
RNU1-1	3.58		
RNVU1-18	3.58		
RNU1-2	3.58		
RNU1-4	3.58		
RNU1-3	3.58		
RNVU1-29	3.58		
AC213203.2	3.58		
CRTC2	3.58		
RIT1	3.56		2.01
ERRFI1	3.51		2.62
HMGCS2	3.46		
TMEM92	3.43		
ITGA3	3.43		
FOXD1	3.39		
PRSS36	3.39		
KLF11	3.36		
UBASH3B	3.36	2.46	
EREG	3.34		
KCNG3	3.34		2.14
MISP	3.34	2.69	
NAMPTP1	3.32		
PTPRS	3.32		
NUAK1	3.29	3.51	
HK2	3.27		
KDM7A	3.25	2.03	
HAMP	3.23		2.57
PALLD	3.23	2.53	
VMAC	3.23		
CYP4F2	3.23		
AL035461.2	3.20		2.31
MAGEH1	3.20	2.83	
CTC-429P9.3	3.20		
SGMS1-AS1	3.20		
GLP2R	3.18		
EPHA2	3.18	2.36	
KDM3A	3.16		
GPAM	3.16		
ENSG00000272153	3.16		
OSBP2	3.16	2.62	
SRGAP3	3.14		3.07
PLCXD3	3.14	4.69	

SYBU	3.12		
AC004908.2	3.10		
ZF530	3.05		
HMGCS1	3.05		
AC026202.2	3.03	3.51	
DLX2	3.03		
COL2A1	2.99	2.22	
LINC01521	2.97		
RSRC2	2.97		
FAM47E-STBD1	2.97		
BAIAP2	2.95		
UGT2B10	2.95	2.83	
ZNF628	2.95		2.60
RASD1	2.93	2	
TMEM151A	2.93		
MRVI1	2.91		
SNX22	2.89		
SEC14L5	2.87		
JUN	2.87		
LONRF3	2.87		
EID2B	2.83		
METRNL	2.83	2.55	
STK17A	2.83		
NR4A1	2.83		
DLX6-AS1	2.81		
CRYM	2.81	2.75	
ARL4C	2.81		
FAM160A1	2.81		
HECW2-AS1	2.81		
DOK1	2.79	2.87	
GRB10	2.79	2.25	
RAB20	2.79		
SLC2A3	2.79		
FRMD5	2.79		
JDP2	2.79	3.66	
ZNF649	2.77		
FGD6	2.73	2.39	
STBD1	2.73		
ASH1L-AS1	2.71		
TANC1	2.7	2.13	
AC012065.3	2.69		
SLC25A47	2.69	2.16	
TMEM147-AS1	2.69		
CSRNP1	2.69		
LNP1	2.68		

LINC01023	2.68		
IL11	2.68	3.39	
GIHCG	2.66		
FERMT2	2.66		
TMCC1	2.66		
DIPK2A	2.64		
ADAM19	2.64		
FAM111B	2.62		
JUNB	2.62		
MXD1	2.62	2.17	
MYOF	2.62		
AL732292.2	2.60		
FBLIM1	2.60	2.14	
ELOVL6	2.58		
LYNX1	2.58		
AL139246.5	2.58		
FAM21FP	2.58		
AL592148.3	2.58		
ANKRD1	2.58		
AKR1B10	2.58	2.97	
DNAJB4	2.58	2.62	
SRSF5	2.57		
RFLNB	2.57		
AP000525.1	2.57		
IDI1	2.57		
NEDD9	2.57		
TENT4A	2.57		
TPSG1	2.57		
TAGLN	2.57		
SERTAD1	2.57		
ZNF37BP	2.55		
ZNF684	2.55		
CBX7	2.55		
INTS13	2.55		
CEBPD	2.55		
LOXL2	2.55		
AGR2	2.55		
FOXPS4-AS1	2.53		
ZNF441	2.53	3.41	
RPL5P11	2.53		
GDF15	2.53	2.04	
DGCR8	2.51		
SNAPC3	2.51		
SHLD2P1	2.50		
SOCS5	2.50	2.07	

WWTR1	2.50		
DNAJB1	2.50	2.25	
AC018647.2	2.48		
IPMK	2.48		
FHL2	2.48		
AL161772.1	2.46		
LYSMD4	2.46		
MORN4	2.46		
BISPR	2.45		2.43
PLEKHA2	2.45		
MEGF8	2.41	2.04	
LINC00957	2.41		
AC068888.1	2.41		
SAMD4A	2.41		
SAP30	2.41		
RLF	2.39		
DUSP8	2.39		
CD19	2.38		
PYCR3	2.38		
GRB7	2.38		
YEATS2	2.38		
RORA	2.38		
SV2A	2.36		
NAB2	2.36		
IL17RE	2.35		
GS1-358P8.4	2.35		
AC004812.2	2.35		
CHIC2	2.35		
WEE1	2.35		
LYPD3	2.33		
LINC01560	2.33		
RDH12	2.33		
EFNA3	2.33		
SPRY4	2.33	2.25	
AP001059.1	2.31	2.69	
LINC00997	2.31		
AC010442.1	2.31		
ACTG1	2.31		
SOS1	2.31		
ENSG00000160180	2.30		
AL356488.3	2.30		
PAWR	2.30		
NBEAL2	2.28		
FAM111A	2.28		
FOXA3	2.28		

MYL9	2.28		
CRABP2	2.28		
BEND3	2.27		
AL161729.1	2.27		
RSBN1	2.27		
FUT11	2.27		
LINC00667	2.25		
CCDC138	2.25		
RYBP	2.25		
ITPRIP	2.25	2.62	
GNB1L	2.25		
NKX2-5	2.25		
AC135050.6	2.23		
DUS2	2.23		
SALL2	2.23		
IL34	2.23		
HILPDA	2.23		2.36
TRAF3IP2-AS1	2.23		
PPP3CC	2.23		
SNORD13	2.23		
AF127577.4	2.23		
GPCPD1	2.22	2.17	
RHEX	2.22		
CLK3	2.22		
NAMPT	2.22		
HIVEP2	2.22		
VGF	2.22		
THAP8	2.22		
PAOX	2.20		
GNA13	2.20		
PELI1	2.20		
FBXO9	2.19		
MSANTD3	2.19		
SP5	2.19		
INHBB	2.19		2.22
PIM1	2.19		
JMJD6	2.19		
MAFK	2.19		
UCA1	2.19		
SLC38A11	2.17		
SIAH2	2.17		
ZYX	2.17		
STC2	2.17		
KDM4C	2.17		
RP11-504P24.8	2.17		

ILF3-DT	2.16		
ZNF654	2.16		
PSMB9	2.16		
JAG1	2.16		
STK26	2.16	2.08	
ZNF100	2.14	2.51	
AL161421.1	2.14		
TCF12	2.14		
GCLC	2.14		
BTBD11	2.14		
EGLN1	2.14		
RP11-517P14.2	2.13		
AL109918.1	2.13		
CAMSAP3	2.13		
DCBLD2	2.13		
PLK2	2.13		
MTCL1	2.13		
ZNF556	2.11		
HHEX	2.11		
UBALD1	2.11		
WTIP	2.11		
SERPINE1	2.11		
FAM98A	2.10		
AMER1	2.10		
HSD11B2	2.10		
U91328.19	2.10		
FDPS	2.10		
EAF1	2.10		
SERTAD2	2.10		
THAP1	2.10		
DEPP1	2.08		
TIGD3	2.08		
ACSM5	2.08		
NR5A2	2.08		
FAM217B	2.07		
AL035071.1	2.07		
LINC00526	2.07		
BRI3BP	2.07	2.04	
SMIM19	2.07		
ZNF213-AS1	2.07		
RP11-1275H24.3	2.07		
SLC25A36	2.07		
TMEM51	2.07		
OSBPL10	2.07		
HUS1	2.07		

OSGIN1	2.07		
MT-CYB	2.06		
GDPD1	2.06		
PWAR6	2.06		
HPCAL1	2.06		
RHPN2	2.06		
PROSER2	2.06		
PIM3	2.06		
SEC24B	2.06		
CPEB4	2.06		
AC012321.1	2.06		
DNAJB9	2.06	2.19	
OXLD1	2.04		
SRSF6	2.04		
AC132872.1	2.04	2.01	
NBPF14	2.04		
SIRT1	2.04		
SH3RF1	2.04		
NFIL3	2.04		
FBXO42	2.04		
PXK	2.04		
FBX08	2.04		
PRDM10	2.04		
ACACA	2.03		
AC012123.1	2.03		
SEC24A	2.03		
BCL10	2.03		
MAPK7	2.03		
PIGW	2.01		
CNTD2	2.01		
AL845472.2	2.01		
AREG	2.01		
CNOT8	2.01		
ANGPTL4	2.01		3.41
MXI1	2.01		
NUDT3	2		
PXMP2	2		
ELMOD2	2		
ZNF331	2		
GPLD1	2		
SMOX	2		
PDK1	2		
ERF	2		
STX3	2		
ARPIN-AP3S2	2		

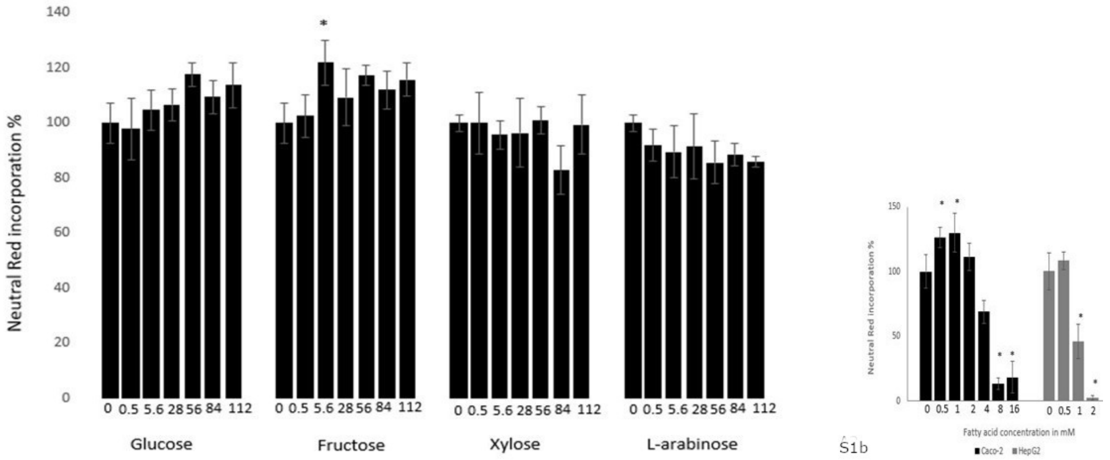

S1a

S1b

Figure S1.

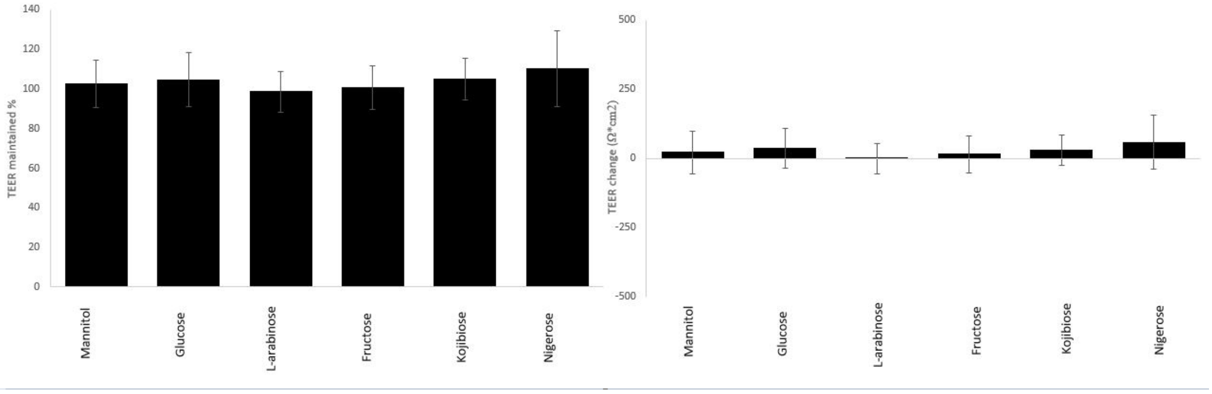

Figure S2.

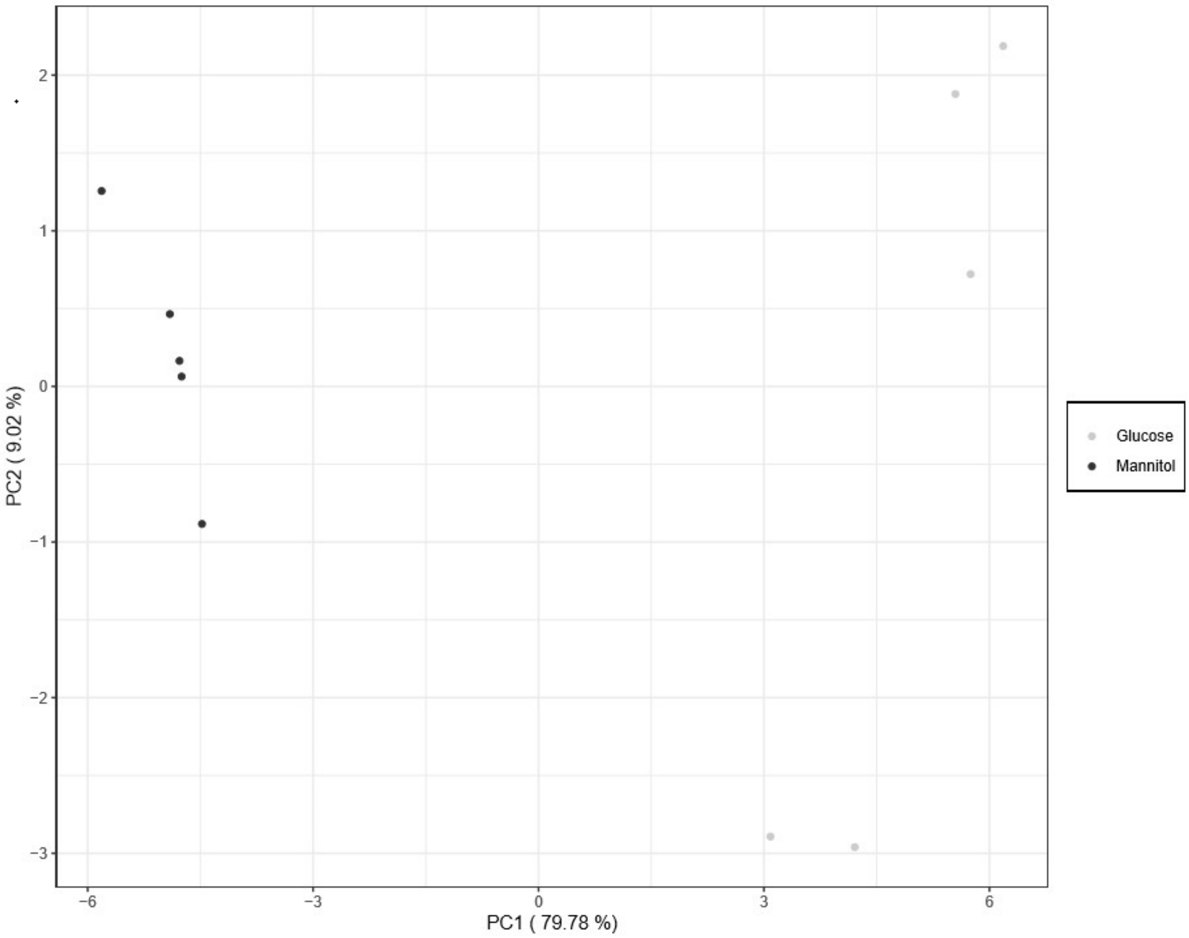

Figure S3a.

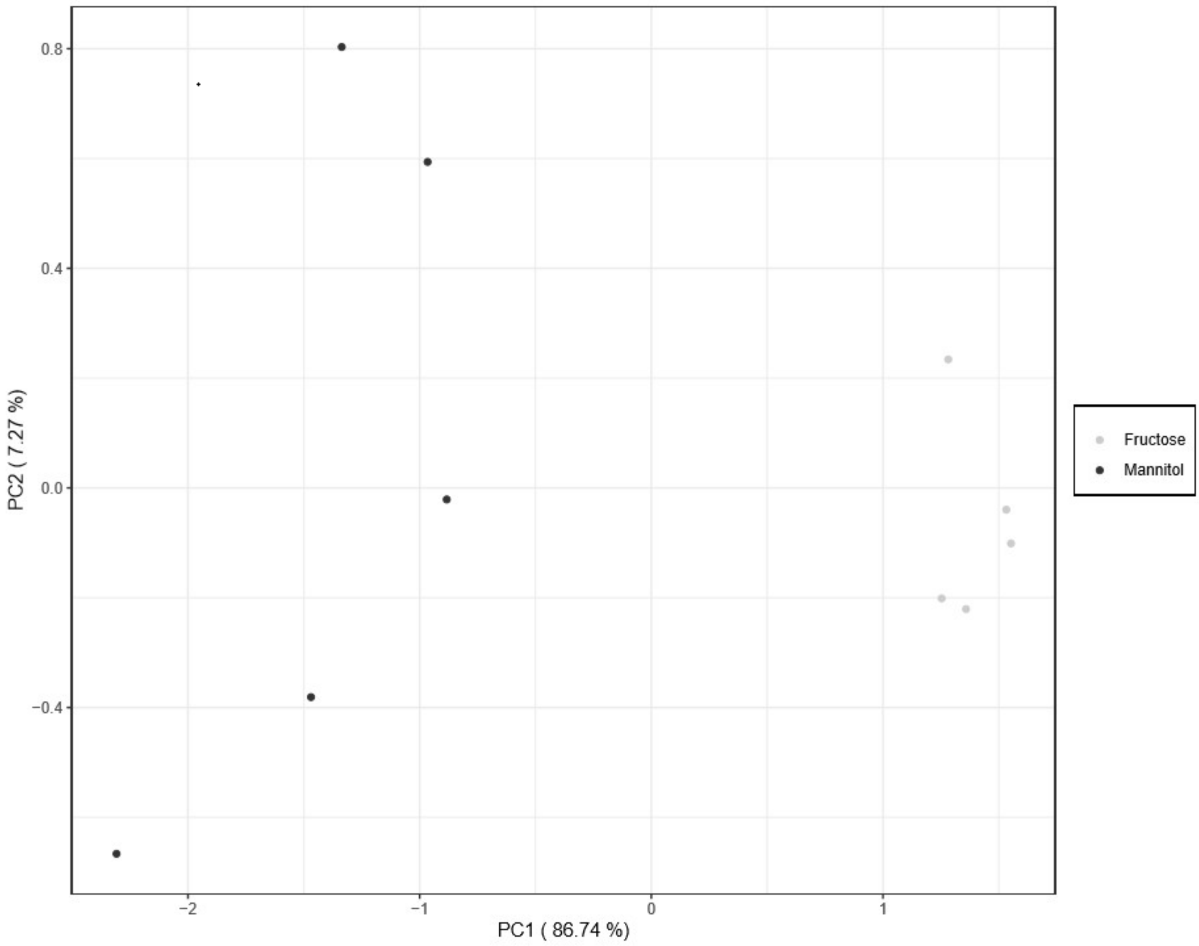

Figure S3b.

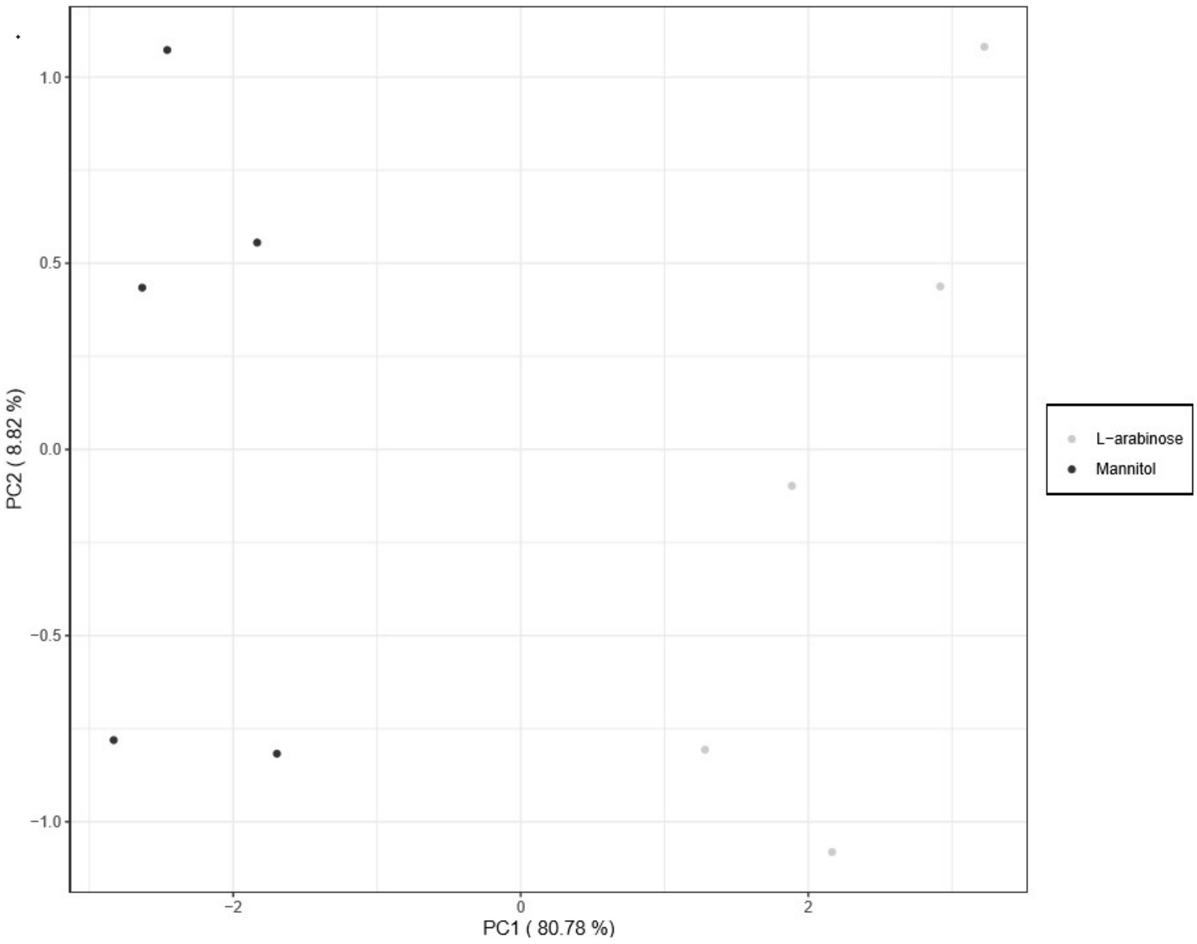

Figure S3c.

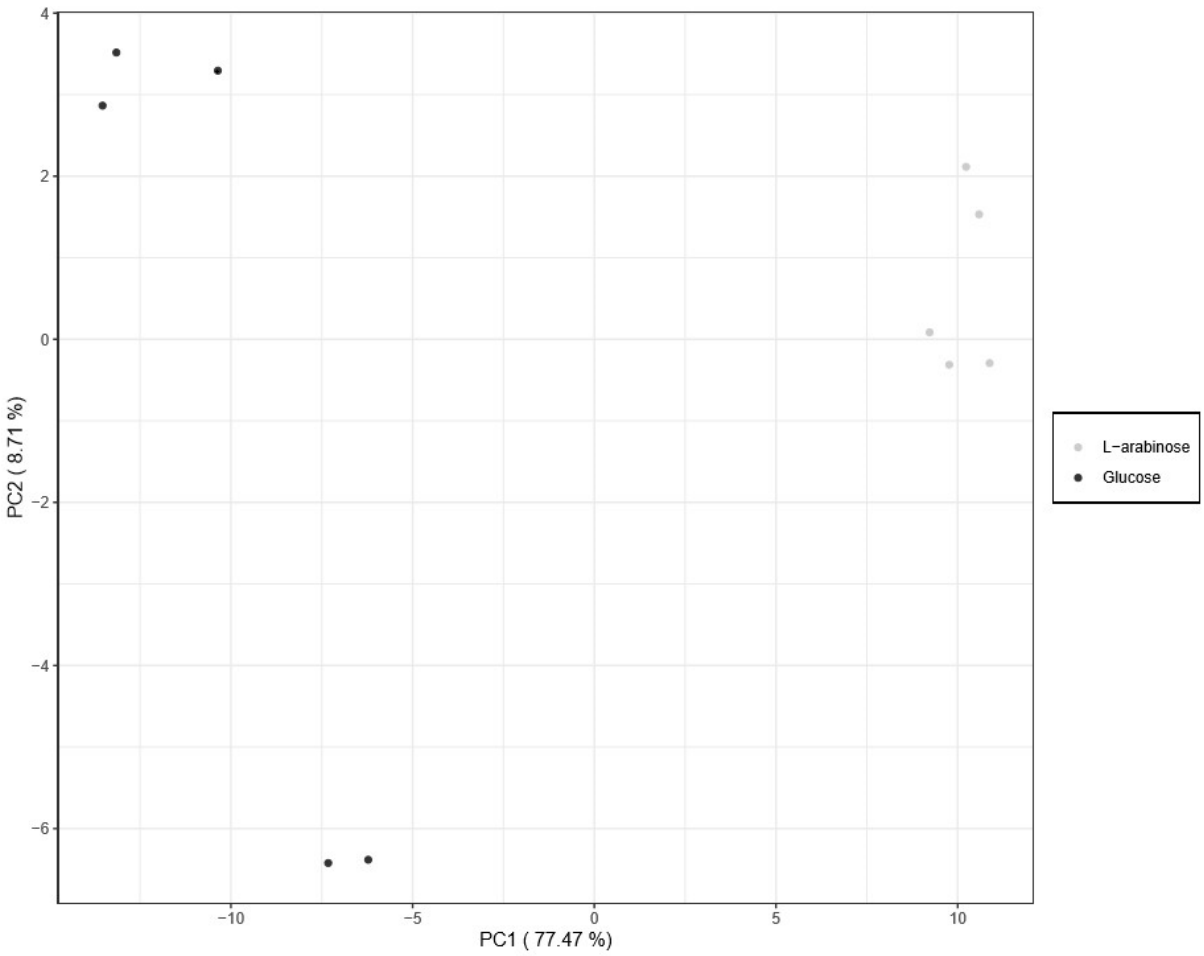

Figure S3d.
